# Understanding the Impact of Stent and Scaffold Material and Strut Design on Coronary Artery Thrombosis from the Basic and Clinical Points of View

**DOI:** 10.3390/bioengineering5030071

**Published:** 2018-09-04

**Authors:** Atsushi Sakamoto, Hiroyuki Jinnouchi, Sho Torii, Renu Virmani, Aloke V. Finn

**Affiliations:** 1 CVPath Institute, Inc., Gaithersburg, MD 20878, USA; asakamoto@cvpath.org (A.S.); hjinnouchi@cvpath.org (H.J.); storii@cvpath.org (S.T.); rvirmani@cvpath.org (R.V.); 2 School of Medicine, University of Maryland, Baltimore, MD 21201, USA

**Keywords:** bioresorbable vascular scaffold, drug eluting stent, polymer, stent thrombosis, scaffold thrombosis

## Abstract

The technology of percutaneous coronary intervention (PCI) is constantly being refined in order to overcome the shortcomings of present day technologies. Even though current generation metallic drug-eluting stents (DES) perform very well in the short-term, concerns still exist about their long-term efficacy. Late clinical complications including late stent thrombosis (ST), restenosis, and neoatherosclerosis still exist and many of these events may be attributed to either the metallic platform and/or the drug and polymer left behind in the arterial wall. To overcome this limitation, the concept of totally bioresorbable vascular scaffolds (BRS) was invented with the idea that by eliminating long-term exposure of the vessel wall to the metal backbone, drug, and polymer, late outcomes would improve. The Absorb-bioabsorbable vascular scaffold (Absorb-BVS) represented the most advanced attempt to make such a device, with thicker struts, greater vessel surface area coverage and less radial force versus contemporary DES. Unfortunately, almost one year after its initial approval by the U.S. Food and Drug Administration, this scaffold was withdrawn from the market due to declining devise utilization driven by the concerns about scaffold thrombosis (ScT) seen in both early and late time points. Additionally, the specific causes of ScT have not yet been fully elucidated. In this review, we discuss the platform, vascular response, and clinical data of past and current metallic coronary stents with the Absorb-BVS and newer generation BRS, concentrating on their material/design and the mechanisms of thrombotic complications from the pre-clinical, pathologic, and clinical viewpoints.

## 1. Introduction

Percutaneous coronary intervention (PCI) using stents continues to be the dominant means by which symptomatic coronary disease is treated. Since PCI was introduced in the mid-1970s, innovation of this technology (e.g., coronary stent and anti-platelet therapy) is constantly being refined to overcome limitations such as in-stent restenosis (i.e., development of drug eluting stent [DES]). Although current day DES have resolved many of the problems of 1st generation DES, including delayed vascular healing and late-stent thrombosis (ST), the long-term durability of these devices is still not optimal. Limitations include the development of neoatherosclerosis, lack of adaptive remodeling due to vessel caging by metal prosthesis, and abnormal coronary vasomotion. To overcome these issues, the concept of totally bioresorbable vascular scaffolds (BRS) was invented with the idea that by eliminating long-term exposure of the vessel wall to drug and polymer, vessel wall physiology and long-term outcomes would improve. However, the most comprehensively tested BRS, the Absorb-bioabsorbable vascular scaffold (Absorb-BVS (Abbott Vascular, Santa Clara, CA, USA), did not live up to its promise as it demonstrated higher events due to greater scaffold thrombosis (ScT) versus a contemporary metallic DES in clinical trials and post-marketing studies, and was subsequently withdrawn from the market in mid-2017 after low commercial sales. Careful analysis of the promises and pitfalls of the Absorb-BVS may allow us to understand how to better design the next generation BRS which has the potential to revolutionize the interventional treatment of coronary artery disease. In this article, we review the design, vascular response, and clinical data of past and current generation metallic coronary stents, as well as the Absorb-BVS and newer generation BRS focusing on the relationship between their material/design and the mechanisms of thrombotic complications from the pre-clinical, pathologic, and clinical viewpoints.

## 2. Metallic Stents

Since the bare metal stent (BMS) was introduced into the clinical arena in 1994 [1,2], several metal alloys have been applied as platform material for metallic stents. There are some properties of metal that make it an attractive material for stents, including appropriate flexibility, radial force, resistance to fracture, radiopacity (especially for chromium [Cr] alloys), biocompatibility, and low thrombogenicity. To date, metallic alloys available for construction of stent platforms include 316L stainless steel (SS), Cobalt Chromium (CoCr), platinum chromium (PtCr), nitinol, and titanium [3,4] (Table 1 and Table 2). Generally, the performance of stents depends on a trade-off between deliverability, reflected in a low-crossing profile, and flexibility (related to both strut thicknesses and the number of connectors between rings) on one hand, and the radial and longitudinal forces exerted by the expanded struts on the other. CoCr and PtCr alloys have higher tensile strength, are more radiopaque than 316L SS, and are currently the predominant material for metallic stents used to treat coronary artery disease. Although a prior pre-clinical study reported the superiority of nitinol versus 316L SS regarding thromboresistant properties [5], no clinical evidence has revealed that a particular metallic platform has superiority over others in terms of biocompatibility and safety. However, it is known that metals such as cobalt, chromium, tungsten, and nickel, all components of today’s coronary stents, can provoke immune reactions. Rare cases of hypersensitivity to metallic stents have been reported [6,7].

Stent strut designs have been classified into open-cell and closed-cell. Open-cell designs have more conformability and deliverability, especially in highly tortuous vessels, while closed-cell have stronger radial force, less recoil, especially in severely calcified lesions, and less plaque distal embolization when they are placed over lipid- or thrombus-rich atherosclerotic lesions [8,9]. These material and design characteristics are important when stents are delivered in diseased human coronary arteries which have complex characteristics (e.g., severely calcified, extremely tortuous, and/or lipid-rich thrombotic lesions).

Prior studies of animal models and human stented arteries clearly show strut thickness has an important impact on medial layer injury and inflammation, with a thicker strut design leading to a higher degree of inflammation and neointimal hyperplasia than thinner strut design [3]. In the ISAR-STEREO randomized clinical trial, the performance of thick-strut BMS (140 µm) was compared with that of a thin-strut BMS (50 µm). The rate of angiographic restenosis at 1 year in the thick-strut group was 25.8% versus 15.0% in the thin-strut group (*p* = 0.003). The rate of re-intervention due to restenosis was 13.8% in the thick-strut group versus 8.6% in thin-strut group (*p* = 0.03) [10]. In pathologic studies of stented human coronary arteries, vascular injury (i.e., trauma of the internal elastic lamina and the medial wall as well the presence of peri-strut inflammation) is a major contributor to in-stent restenosis (ISR) associated with BMS [11]. Moreover, our series of 59 autopsy cases who died within 30 days of PCI for acute coronary syndrome revealed the pathologic risk factors for early ST [12]. The percentage of necrotic core prolapse, medial tear, or incomplete stent apposition was significantly higher in early ST cases (*n* = 34) than in patent stents (*n* = 25) (28% versus 11%, *p* < 0.001; 27% versus 15%, *p* = 0.004; and 34% versus 18%, *p* = 0.008, respectively). Multivariate analysis indicated maximal depth of strut penetration, % struts with medial tear, and % struts with incomplete apposition were the primary indicators of early ST [12]. Thus, thick-strut stents are more likely to induce severe injury in the atherosclerotic vasculature and to have more thrombogenicity versus thin-strut stents.

**Figure 1:**
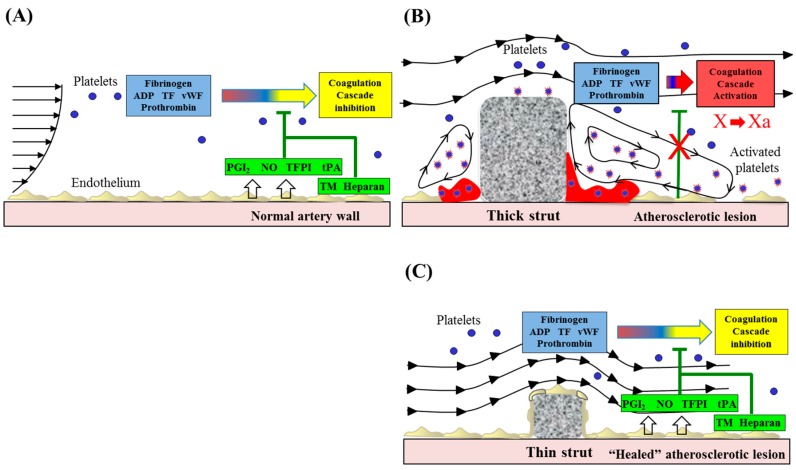
Alteration of blood flow dynamics and thrombogenicity in the vicinity of thin or thick stent struts. Stent-induced flow disturbances affect thrombogenicity and re-endothelialization following stent implantation. (**A**) Healthy endothelium in the normal artery wall expresses anticoagulant and antithrombotic molecules, including NO, PGI2, TFPI, tPA, TM, and heparin-like molecules. (**B**) Stent placement results in local endothelial denudation, which leads to activation of the coagulation cascade. Stents, especially thicker strutted ones, may promote non-streamlined flow separation in regions proximal and distal to struts. Accelerated blood flow (high shear) over the strut edges can activate platelets through the release of thromboxane A2 and ADP, whereas flow recirculation zones with low shear rates are associated with inhibition of re-endothelialization, potentially enabling procoagulant and proinflammatory elements to accumulate, which contribute to thrombus formation. (**C**) A thin strut geometry reduces flow separation and low shear, leading to the inhibition of platelet activation. Moreover, the generation of recirculation zones proximal and distal to thin struts will be minimized, resulting in the reduced thrombogenicity. Undisturbed flow proximal and distal to streamlined struts promotes re-endothelialization, which further helps to maintain hemostatic balance and prevent thrombosis. Modified and reprinted with permission from Jimenez, J.M.; et al. Ann. Biomed. Eng. 2009 [14]. ADP: adenosine diphosphate, AR: aspect ratio, CFD: computed flow dynamics, NO: nitric oxide, PGI2: prostacyclin, TF: tissue factor, TFPI: tissue factor pathway inhibitor, TM: thrombomodulin, tPA: tissue plasminogen activator, vWF: von Willebrand factor.

Consistent with the results of clinical and pathologic studies, basic pre-clinical data using computational fluid dynamics showed that excessive stent strut density and greater thickness of struts increased local blood flow disturbances contributing to thrombogenicity [13,14]. There are platelet activation and enhancement of other coagulation factors in the proximal and distal portion of stent struts in areas of blood-flow recirculating zones (Figure 1). Kolandaivelu et al. reported using an ex vivo shunt model, thick struts (162 μm) increased thrombogenicity 1.5-fold as compared to thin struts (81 μm) [13]. Thick-strut stents also showed a 1.6-fold greater thrombus coverage versus thin-struts (*p* = 0.004) in 3-day post-implantation porcine coronary arteries [13]. Furthermore, Jimenez and Davies compared flow dynamics between rectangular versus circular strut stent configurations and showed that the former have larger recirculating zones and lower endothelial regrowth, with greater effects on the downstream versus upstream areas [14]. The authors showed that pro-coagulant conditions are greatly increased around stent struts when there are conditions such as accelerated flow over the strut edges, which results in greater shear stress that leads to activation of platelets. In addition, platelet retention in recirculating zones together with pro-coagulant factors may reach a critical concentration for accelerating clot formation. De-endothelialization during stenting and balloon angioplasty removes naturally expressed anti-coagulants in the endothelium and exposes a subendothelial matrix predisposed towards thrombosis. Lastly, low shear stress around large struts also inhibits re-endothelialization [14] (Figure 1). Additionally, the rate of endothelial cell coverage of stent struts is also greatly affected by their thickness. Palmatz et al. showed, using their in vitro flow chamber model at physiologic wall shear stress (15 dynes/cm^2^), that obstacle heights of 100 μm or greater had significantly less coverage of endothelial cells than those which were 25 μm [15].

## 3. Drug and Polymer

**Figure 2:**
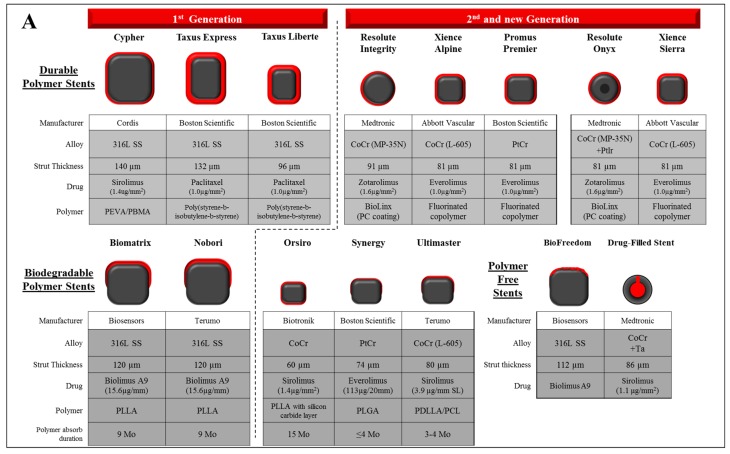

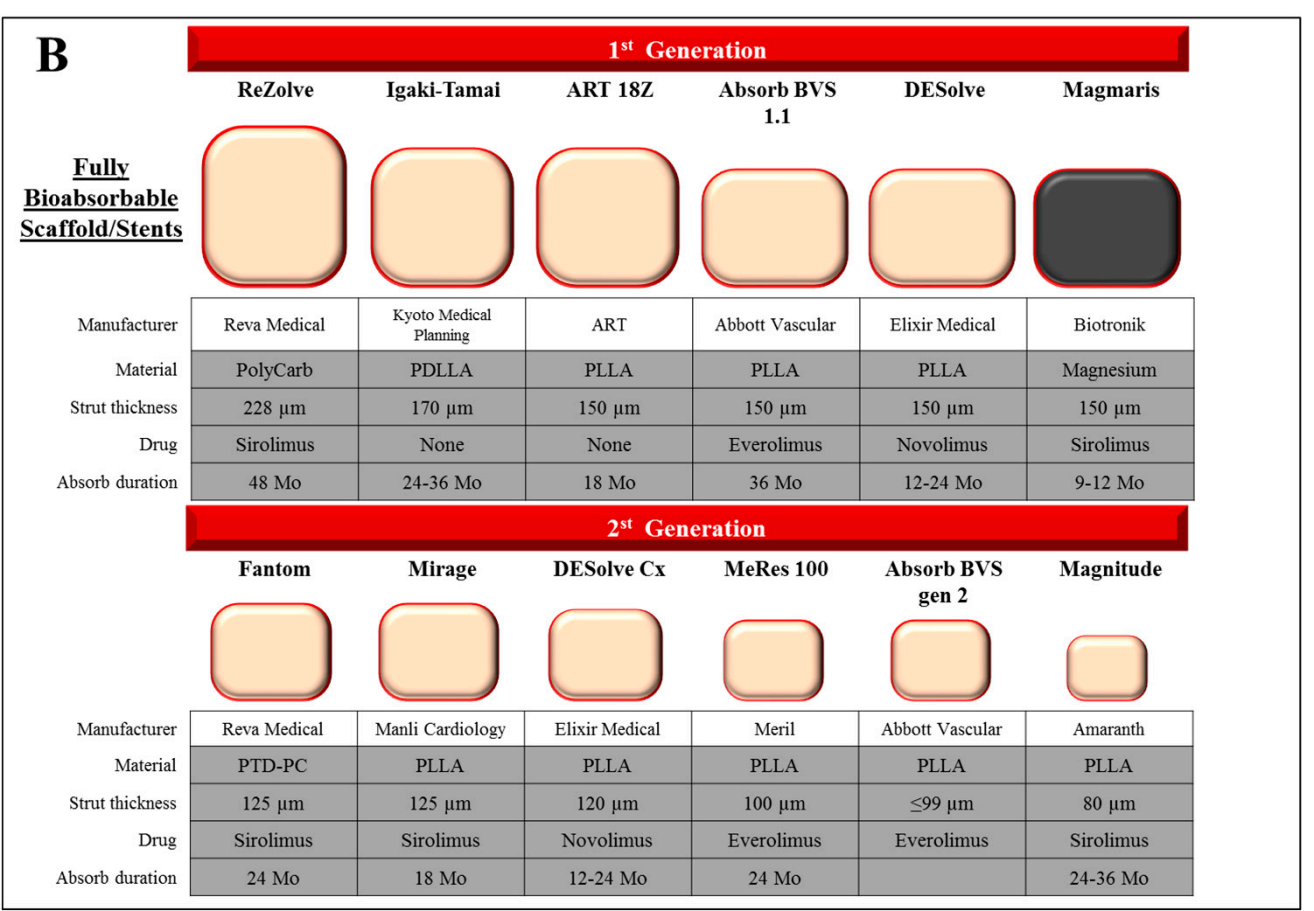
Design characteristics of representative drug eluting stent and bioabsorbable scaffold/stent. (**A**) The characteristics of past and current commercial drug-eluting stents including durable polymer (DP)-, biodegradable polymer (BP)-, and polymer free-DES. Types of materials (alloy, drug, and polymer), strut thickness, and estimated duration for polymer absorption (in BP-DES) of each stent are described. (**B**) The characteristics of 1st and 2nd generation fully bioabsorbable scaffold/stents. Types of materials (polymer, alloy, and drug), strut thickness, and estimated duration for polymer absorption period (in BP-DES) of each stent were described. Co: cobalt, Cr: chromium, Ir: iridium, Mo: months, PBMA: poly(butyl methacrylate), PCL: poly-*ε*-caprolactone, PC: phosphorylcholine-coated, PDLA: poly-d-lactic acid, PDLLA: poly-d,l-lactic acid, PDLGA: poly(d,l-lactide-*co*-glycolide), PEVA: poly (ethylene-vinyl acetate), PGA: polyglycolic acid, PLLA: poly-l-lactic acid, PLGA: poly(lactide-*co*-glycolide), PolyCarb: poly-tyrosine-derived polycarbonate polymer, Pt: platinum, PTD-PC: polytyrosine-derived polycarbonate, SS: stainless steel, Ta: tantalum.

Since neointimal overgrowth is the main cause of ISR, its inhibition is the primary mechanism to achieve better clinical outcomes—mainly lower rates of target lesion revascularization (TLR). To ensure the preservation of healthy endothelial growth and function, the ideal DES should have two different, sometimes conflicting roles: i.e., adequate inhibition of vascular smooth muscle cells proliferation as well as less destruction of endothelial cells. To date, two different classes of drug, i.e., sirolimus and its derivative (mammalian target of rapamycin [mTOR] inhibitors) and paclitaxel (microtubule inhibitor), were applied as anti-proliferative DES drugs. Based on the large body of clinical evidence which demonstrates the superiority of sirolimus-eluting stent (SES) versus paclitaxel-eluting stent (PES) on anti-restenotic efficacy and safety [16], rapamycin and its analogues are currently the major anti-proliferative agents used in coronary DES. Manipulation of drug release kinetics to achieve therapeutic drug levels in the arterial wall for 30–90 days is essential for achieving inhibition of neointimal formation (Figure 2A). For this purpose, polymer-based coatings are largely used in most DES. Polymer drug reservoirs should share the following characteristics: (i) be biocompatible, (ii) do not interact with the active drug, (iii) provide a platform for appropriate drug-eluting kinetics, (iv) behave in a biologically inert manner after the drug has been completely eluted, and (v) be mechanically stable over the long-term in the dynamics of coronary circulation milieu [17]. However, the durable polymer (DP) used in 1st-generation SES, poly-(*n*)-butyl methacrylate, was considered a trigger for late clinical adverse events via chronic hypersensitivity, demonstrating that other polymers do not have these characteristics. An in vitro experimental study supported the concern that degraded methacrylate acid, which is considered bio-stable, enhanced vascular smooth muscle cell apoptosis [18], which might delay endothelial functional recovery and disturb vascular healing. This drawback of the polymer remaining on the vessel wall for a long duration provided the impetus for better biocompatible DP and biodegradable polymer (BP) coatings for metallic stents (Figure 2A).

Strut design is also known to affect drug distribution and anti-proliferative properties on the atherosclerotic arterial wall. While eluted drugs ideally need to be uniformly distributed on the vessel wall, uneven distribution of drugs can be expected in complex coronary lesions, such as those with severe calcification and extreme tortuosity. In this regard, although the deliverability is feasible, a larger open-cell design is more likely to lead to heterogeneous drug distribution within the vessel wall, possibly because of uneven strut spacing which could potentially cause a higher rate of restenosis [19].

## 4. Stent Thrombosis in Metallic Stent

ST is divided into 4 categories determined by the time period at which it occurred after PCI, i.e., acute (<24 h), sub-acute (24 h–30 days), late (30 days–1 year), and very-late (>1 year). Most early ST (<30 days) results from procedural factors involving under-expansion, dissection at the stent edge, plaque rupture in the residual atherosclerotic lesion, and medial layer injury. Therefore, the frequency of early ST is reported as similar between BMS and DES. In contrast, the mechanisms of late and very late ST are related to their materials including drug and polymer. Large-scale clinical data demonstrated a higher frequency of late and very late ST in 1st generation SES and PES, e.g., Cypher (Cordis, Johnson & Johnson, Miami Lakes, FL, USA) and Taxus (Boston Scientific Corp, Natick, MA, USA), versus BMS [20,21]. Ten year follow-up data of patients who were implanted with 1st generation SES and PES (*n* = 2098) in SORT OUT II trial showed that definite, probable, and possible ST appeared in 279 patients (13.3%) with no difference between stent types and with a steady annual rate of 1.3% after the first year [22]. Pre-clinical animal studies using SES and PES implants revealed a reduction of neointimal overgrowth along with less intimal cell proliferation, as well as incomplete vascular healing, especially for PES, which was described as a local toxic effect. Intimal hemorrhage, large fibrin deposition, inflammatory reaction in intima and adventitia, and medial wall necrosis were more likely to be seen at high doses [23,24,25,26]. Additionally, as mentioned above, the pathologic findings in human and animal coronary arteries suggested a polymer-induced chronic hypersensitivity vasculitis, a potential cause of late and very late ST in 1st generation SES [27,28]. Our 81 human autopsy case series of late ST (>30 days) who were implanted with 1st generation DES (Cypher and Taxus) demonstrated that delayed endothelialization at the stented site was the best predictor of thrombosis [29]. These clinical and pathologic studies unearthed major problems with 1st generation DES. Second generation DES, e.g., CoCr-everolimus eluting stent (CoCr-EES) and Xience (Abbott Vascular, Santa Clara, CA, USA), had a more controlled anti-proliferative drug elution profile, a more biocompatible polymer and demonstrated dramatically reduced rates of late ST to a similar or lower level than BMS [30].

Stent fracture (SF) after DES implantation is another contributor to late ST [31,32]. Our prior data from 177 consecutive autopsy cases, including 1st generation SES and PES, showed a higher rate of SF (29%) than what had been reported clinically [33], likely because of the limited sensitivity of angiography for detecting SF. In this cohort, grade-V severe SF (multiple strut fractures with acquired transection with gap in the stent body) was associated with an increased incidence of adverse pathologic findings including thrombosis and restenosis. Although it is not fully understood why SFs cause adverse events, the lack of stent performance, e.g., distortion or acquired under expansion, may play a critical role. In addition, 1st generation SES usage (thick strut (140 μm) with closed cell design), longer implant duration and longer stent length were found to be independent risk factors, suggesting greater metal fatigue causes SF [33].

In addition to SF, emerging evidence suggested that plaque rupture at the site of in-stent atherosclerosis, so called “neoatherosclerosis,” is also one of the major causes of late and/or very late ST. In 2009, we initially reported the neoatherosclerosis in both BMS and 1st-generation DES from human autopsy cases with previous history of PCI [34]. The incidence and initial development of neoatherosclerosis were significantly greater and occurred earlier in DES versus BMS (35% versus 10% and 4 months versus 2 years, respectively). Further, there were no significant differences in the incidence of neoatherosclerosis between 1st and 2nd generation DES (SES 35%, PES 19%, and CoCr-EES 29%) [34]. We believe that anti-proliferative mTOR inhibitors loaded onto stents are one of the primary causes of neoatherosclerosis in DES [35]. In general, healthy vascular endothelial cells play a key role in preventing leukocyte invasion, a critical step of atherogenesis, and platelet aggregation by actively controlling permeability [36]. Endogenous agents, such as histamine, thrombin, and other acute inflammatory mediators increase vascular permeability through alterations in the function and organization of adherens junctions in endothelial cells [36]. A previous pre-clinical study we conducted revealed that mTOR inhibitors induce calcium dependent activation of protein kinase C-alpha, which subsequently disrupts the interaction of endothelial adherens junctional protein complexes (i.e., p120-catenin/vascular endothelial cadherin) on endothelial cells, increasing their permeability [35]. In the rabbit iliac model of stenting, durable polymer DES demonstrated impaired endothelial permeability and greater predisposition to neoatherosclerosis after low cholesterol diet feeding as compared to bare metal stents [37]. In this regard, biodegradable polymer (BP-DES), in which polymer absorbs over time and therefore stops functioning as a drug reservoir within months after initial implantation, demonstrates improved endothelial function as compared to DP-DES [38]. Theoretically, BP-DES should have less late-clinical adverse events related to neoatherosclerosis compared to DP-DES if these findings in animal models also occur in humans. While several randomized clinical trials compared BP-DES with DP-DES, they did not initially show a clear advantage for BP-DES [39]; more recent data suggest an advantage for bioabsorbable polymer Synergy and Orsiro stents versus DP-DES [40,41].

## 5. Bioabsorbable Scaffold and Stent

Even though the technological developments in metallic DES improved clinical outcomes, permanent metal implants still have intrinsic disadvantages, including stent fracture, allergic reaction to the metal and/or polymer and the risk of late restenosis and late ST. In fact, the long-term outcome of 2nd-generation durable-polymer DES regarding death and non-fatal myocardial infarction (MI) was not different from that of BMS, and clinical events continue to occur in BMS as well as DES [42]. Moreover, metallic caging could potentially preclude further intervention and/or bypass surgery. Thus, the concept of a totally bioabsorbable scaffold, which would theoretically help improve the recovery of vascular function, has fascinated scientists and interventional cardiologists. The ideal material for this concept might have appropriate temporal radial strength to resist recoil, flexibility, biocompatibility and resorb without massive inflammatory reaction or systemic toxicities. Thus far, there are two major categories of material which are used in bioabsorbable scaffold/stent: polymer and bioerodible metallic alloy (Table 2, Figure 2B).

## 6. Polymer Scaffold

There are different polymer chain arrangements—e.g., linear, branched, or cross-linked [43]. A polymer’s crystallinity or amorphous nature determines its strength and degradation rate. In general, the greater the molecular weight (i.e., longer chain of monomers), the greater the strength and the longer absorption time the polymer will have [44]. Polylactic acid (PLA) is the most common biodegradable polymer used for vascular scaffolds. There are several different types of PLA such as Poly-l-lactic Acid (PLLA), Poly-d-lactic Acid (PDLA), and Poly-d,l-lactic Acid (PDLLA). PLLA and PDLA are rigid, transparent polymers which generally come in a semi-crystalline form containing both a very ordered crystalline-chain structure interrelated with random amorphous chains which are more susceptible to hydration. In contrast, copolymer PDLLA is an amorphous polymer [45,46]. Polyglycolic acid (PGA) is highly crystalline and less hydrophobic than PLA. Because of its high degradation speed, PGA is generally prepared with PLA or poly-ε-caprolactone (PLC) as copolymer poly(lactide-*co*-glycolide) (PLGA) or poly(glycolide-*co*-caprolactone) (PGCL) [45]. PLC is semi-crystalline and has high flexibility and elasticity (Table 2). Although the mechanical characteristics of polymers can be manipulated, it is still challenging to create a scaffold with equivalent radial strength and flexibility to that of a metallic stent. Thus, most polymer-based scaffolds are bulkier than currently available metallic stents in order to improve strength.

The mechanisms of in-vivo polymer degradation include mechanical, thermal, and hydrolytic processes. In hydrolysis, polymers reduce their molecular weight by reaction with water molecules and crack into oligomers and monomers [47]. The residues go through phagocytosis by macrophages and are metabolized into water and carbon dioxide. This process is generally accompanied by an inflammatory reaction consisting of macrophages, giant cells, and lymphocytes.

## 7. Absorb-BVS: A Major Concern of Scaffold Thrombosis from Pathologic Viewpoint

Absorb-BVS, a non-metallic scaffold made of PLLA backbone coated with PDLA and PLLA, with everolimus with a total strut thickness of 157 μm (strut + polymer), was the most widely utilized BRS. Despite its thicker struts compared to metallic stents, it had lower tensile strength and stiffness, limited elongation, lower mechanical strength, and lower ductility. The increased crossing profile and limited mechanical properties of this product required further attention during delivery and deployment (i.e., aggressive pre-dilatation, precise vessel sizing and limited post-dilation diameter as compared with current metallic DES).

The initial 1-year results for Absorb III large scale randomized trial evaluating clinical outcomes of Absorb-BVS (*n* = 1322) and CoCr-EES (*n* = 666) involved about 70% of patients with stable ischemic heart disease and showed non-inferiority for Absorb-BVS with respect to cardiac death (0.6% versus 0.1%, respectively; *p* = 0.29), target-vessel MI (6.0% versus 4.6%; *p* = 0.18), ischemia-driven TLR (3.0% versus 2.5%; *p* = 0.50), and device thrombosis (1.5% versus 0.7%; *p* = 0.13) [48]. Nevertheless, the rate of clinical adverse events increased beyond one year of follow-up. In the AIDA trial, in which around 40% of patients had stable ischemic heart disease, there was a higher rate of ST (3.4%) in Absorb-BVS than in metallic DES (0.9%, relative risk of 3.86, *p* < 0.001) at 2 years [49]. A recent meta-analysis of Absorb-BVS versus metallic EES including 5583 patients from seven randomized clinical trials (Absorb-BVS: *n* = 3261, metallic EES: *n* = 2322) reported definitively higher 2-year relative risk for Absorb-BVS in terms of device-oriented composite endpoint versus EES (9.4% [304/3217] versus 7.4% [169/2299], relative risk [RR] 1.29 [95% confidence interval (CI) 1.08–1.56], *p* = 0.0059). The differences were mainly driven by increased target vessel-related MI (5.8% in Absorb-BVS versus 3.2% in EES; RR 1.68 [95% CI 1.29–2.19], *p* = 0.0003) and ischemia-driven target lesion revascularization (5.3% in Absorb-BVS versus 3.9% in EES; RR 1.40 [95% CI 1.09–1.80], *p* = 0.009) [50]. Moreover, Absorb-BVS showed a higher cumulative incidence of ScT at 2-years versus EES (2.3% in Absorb-BVS versus 0.7% in EES; RR 3.35 [95% CI 1.96–5.72], *p* < 0.0001).

**Figure 3:**
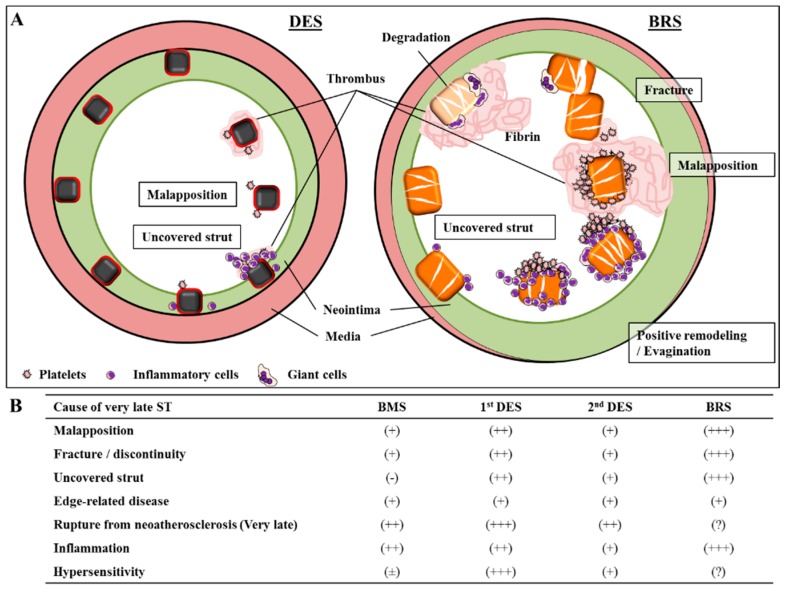
Various mechanisms of very late stent (ST) and scaffold thrombosis (ScT). (**A**) Diagram illustrating the various mechanisms of very late stent (ST) (left) and scaffold thrombosis (ScT) (right). (**B**) Impact of each factor and its relationship to ST and ScT in different devices—BMS = bare metal stents, DES = drug-eluting stents, BRS = bioresorbable vascular scaffold. Permission obtained from Mori, H; et al. Coron. Artery Dis. 2017 [54].

A small reference vessel diameter (<2.25 mm) was an independent predictor of adverse clinical outcome in Absorb-BVS [50]. Appropriate implantation technique was proposed as a method to reduce ScT. From their multi-center observational registry, Puricel et al. reported the causal relationship between the risk of ScT and inadequate procedural techniques during device placement such as pre-dilatation, sizing, and post-dilatation [51]. Nevertheless, it seems clear based upon the particular characteristics of the Absorb-BVS discussed below that not all ScT can be prevented by adapting the proper device implant technique and sizing (Figure 3). Different mechanisms for scaffold thromboses within and beyond 1 year have been proposed from prior clinical data analyses. In cases of ≤1-year device implantation, thrombotic events were largely related to the thick strut device being placed into small vessels (reference vessel diameter <2.40 mm) [52], whereas scaffold thromboses >1-year following deployment were mainly due to intraluminal scaffold dismantling due to discontinuity of uncovered scaffold strut [53].

Recently, the mechanisms of very late ScT in Absorb-BVS were analyzed via optical coherence tomography (OCT) in a large clinical trial in Europe. Yamaji et al. reported 36 cases with 38 lesions of very late ScT who underwent OCT assessment during urgent revascularization at 19 centers [53]. The causes of very late ScT and its frequency were classified as follows: scaffold discontinuity (41%), malapposition (18.4%), neoatherosclerosis (18.4%), under expansion or scaffold recoil (10.5%), uncovered struts (5.3%), and edge-related disease progression (2.6%) [53]. Even though OCT has limitations for describing some of these phenomena (e.g., mistaking struts with attached fibrin/thrombus as endothelialized covered struts) these findings suggest some of the underlying pathologic mechanisms for ScT, made even more important because of the lack of large autopsy data.

**Figure 4:**
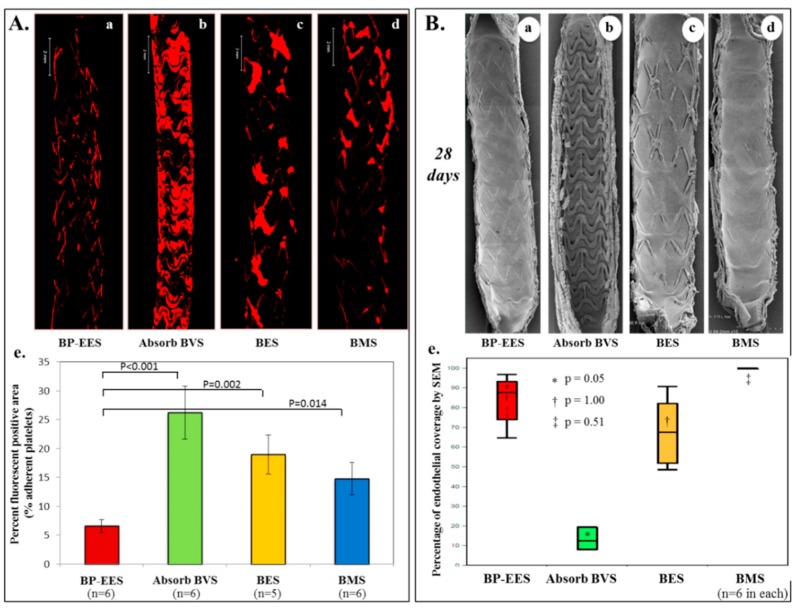
Acute thrombogenicity and delayed endothelial coverage of Absorb-BVS in experimental models. (**A**) Ex-vivo arteriovenous porcine shunt model. Representative images derived from confocal microscopy after 1 h in a swine ex-vivo shunt model. Platelets were stained with anti-CD61 and CD42b primary antibody and red-fluorescent secondary antibody. (a) Biodegradable polymer everolimus-eluting stent (BP-EES); (b) fully bioabsorbable everolimus-eluting scaffold (Absorb-BVS); (c) biodegradable polymer biolimus-eluting stent (BES); and (d) bare metal stent (BMS). (e) Percent fluorescent positive area based on percentage of fluorescent positive staining against CD61 and CD42b within the entire stented segment. Values are expressed as mean ± SD in each group. BP-EES, Absorb-BVS, and BMS included *n* = 6 stents in each group, whereas BES included *n* = 5 stents. One BES was incompletely expanded and therefore excluded in the analysis. (**B**) Rabbit model. Representative images of endothelial coverage assessed by scanning electron microscopy (SEM) at 28 days. Low magnification (15×) SEM images provide an overview of the luminal surface of the bisected segment from proximal (top) to distal (bottom). (a) BP-EES, (b) Absorb-BVS, (c) BES, and (d) BMS. (e) Relative percentage of endothelial coverage above struts assessed by scanning electron microscopy is shown in box and whisker diagrams on the right. Values represent median with lower (25th percentile) and upper quartiles (75th percentile) and whiskers for minimum and maximum value. Each group has *n* = 6, respectively. Modified reprinted from Koppara, T; et al. Circ. Cardiovasc. Interv. 2015 [57].

Fracture or discontinuity of scaffold struts has been commonly reported with Absorb-BVS (25% of lesions at 2 years and 42% at 3 years) [55]. This phenomenon increases with the passage of time after implantation. Discontinuity does not mean the normal absorption process of the polymer, but the dismantling of the scaffold structure due to the mechanical fragility. It is defined as “isolated malapposed struts that cannot be integrated in the expected circularity of the device in at least one cross-section or those with an abrupt loss of longitudinal scaffold between adjacent 2 frames” [56]. Late scaffold disruption and disintegration may occur safely when struts are covered by neointima; in such case, the disrupted strut is trapped with surrounding tissues and has been isolated from circulating blood. Nevertheless, if neointimal coverage of scaffold struts is delayed, the fractured struts will protrude into the vessel lumen, causing blood-flow turbulence and creating a substrate for thrombosis [56]. The strut thickness of this device (157 μm) has an innate disadvantage for endothelialization because of its sheet size and surface area coverage (Figure 4A,B). Additionally, diseased human coronary arterial wall involving necrotic core and severe calcification is likely to further delay endothelial regeneration, which would not be seen in healthy animal model studies required before initial use in humans. In this situation, the thrombotic complication due to mechanical fracture would be more likely.

## 8. Vascular Response to Absorb-BVS

As mentioned above, the thick struts disrupt laminar blood flow and cause recirculating zones which lead to higher thrombogenicity. Additionally, because of its strut thickness, Absorb-BVS struts occupy on average 27% of the vessel wall, whereas most metallic DES occupy only 13%, which itself causes significant flow disturbances, especially in smaller vessels [14]. Furthermore, relatively aggressive pre- and post-dilatation at the site of scaffold implantation might be performed with the intention to achieve complete strut expansion and avoid strut malapposition. But this type of procedure potentially causes more arterial wall damage, exposing sub-endothelial collagen which initiates coagulation cascade via binding to platelet [58] and pro-coagulation tissue factor within the necrotic core [59] to blood stream factors. These device features and implantation procedures may directly and/or indirectly elevate the thrombogenicity at the site of scaffold. On the basis of our pre-clinical view point, malapposition and uncovered struts seem the major cause of ST in both metallic DES and BVS. Moreover, the impact of malapposition and uncovered struts would be much greater in Absorb-BVS than that of contemporary metallic DES [53,60], and would require a longer duration of dual antiplatelet therapy in order to compensate for the much longer healing time.

Late acquired coronary evagination between strut, initially highlighted as a risk factor for late ST in 1st-generation SES era, was also reported in patients receiving Absorb-BVS. Gori et al. analyzed the OCT and angiographic follow-up data of 90 cases (102 scaffolds) that underwent Absorb-BVS deployment [61]. As a result, more than half (54%) of the BVS cases had at least 1 or more evaginations at 12-months follow-up; evaginations strongly were associated with the presence of malapposition and strut fracture. Peri-strut contrast staining was found in 18% of cases. Since evagination and peri-strut contrast staining in 1st-generation SES were thought to result from a chronic inflammatory allergic response, this finding suggested that Absorb-BVS potentially causes inflammatory reaction during polymer degrading process. Although this device is no longer commercially available, careful follow-up of patients receiving Absorb-BVS seems to be needed.

One of the major selling points of Absorb-BVS was the fact that at the end of degradation, the vessel would return to its native state with preserved vasomotion/vasodilation and protection from neoatherosclerosis. Although very long-term follow-up data from Absorb-BVS is limited, several reports of neoatherosclerosis in BVS are gradually accumulating. Most recently, Moriyama et al. analyzed 5-year follow-up data of BVS including OCT assessment in 20 patients (22 lesions) [62]. At 1 and 5 years serial OCT follow-up, significant differences in the prevalence of in-scaffold lipid-laden neointima (17% versus 61%; *p* = 0.04), calcification (28% versus 94%; *p* < 0.01), neovascularization (6% versus 78%; *p* < 0.01), and thin-cap fibroatheroma (0% versus 22%; *p* = 0.02) were observed. Moreover, in non-scaffold native vessel segment, there were no significant differences in plaque prevalence between 1 and 5 years [62]. Even though the number of cases was relatively small, this serial observation demonstrates important insights into the short- and long-terms response to Absorb-BVS. This data is not surprising since, as previously discussed, vascular endothelial permeability is a key pathophysiological alteration in the development of neoatherosclerosis and is augmented by anti-proliferatives such as rapamycin or its analogues loaded into DES [35,37]. Although the initial concept of allowing the vessel to return to its native state does apply to BRS, the relative long amount of time it takes for the Absorb-BVS to be fully absorbed (i.e., 36–42 months in animals) allows plenty of time for the development of neoatherosclerosis. Thus, neoatherosclerosis should be entertained as a cause of late events in patients receiving Absorb-BVS and long-term follow-up should be conducted in these patients.

Another highly anticipated benefit of Absorb-BVS was the return of endothelium-dependent coronary vasodilation, known to be linked to vascular health [63]. Several early clinical studies in patients with stable coronary disease suggested recovery of endothelium-dependent vasomotion assessed by acetylcholine intra-coronary infusion at 1 and 2 year after Absorb-BVS implantation [64,65,66], although the number of allocated patients was relatively small. However, at 3-year follow-up of the Absorb II trial, vasomotor reactivity was not statistically different between Absorb-BVS and CoCr-EES [67]. Four-year follow-up did not report the results of acetylcholine-derived vasodilatation [68]. Recent sub-analysis data of 3 year follow-up from the TROFI II trial, which compared the performance of Absorb-BVS versus CoCr EES in patients with ST-segment elevation MI, did not show any benefit of Absorb-BVS regarding endothelium-dependent vasoreactivity [69]. According to these data, no evidence suggests a clear benefit for return of endothelial dependent vasodilatation in Absorb-BVS. Further work is needed to fully understand and confirm these results.

To date, several bioabsorbable scaffolds based on polymers are in development involving the DESolve novolimus-eluting scaffold (Elixir, Milpitas, CA, USA), and MeRes100 (Meril, Princeton, NJ, USA), Fantom (Reva Medical, San Diego, CA, USA), and Fortitude, Magnitude, and Aptitude (Amaranth, Mountain View, CA, USA), and all of which are limus-eluting scaffolds (Figure 2B). In order for polymer based BRS to be successful, a reduction of the strut profile while maintaining radial strength needs to be demonstrated.

## 9. Bioerodible Metallic Alloy

Although the most widely used material for BRS is polymers such as PLLA, bioerodible metallic alloys are also being investigated. One of the most advanced systems is the magnesium resorbable scaffold (BIOTRONIK AG, Buelach, Switzerland).

Magnesium is a biocompatible metal abundantly contained in the body which is also known as an essential element for normal biological activity in processes such as bone formation, immune system functionality, maintaining muscle and nerve function, and anti-arrhythmic effect in heart. The amount of released magnesium from currently developed stents has almost no effects on the plasma concentration. The resorption process of magnesium alloy has two different phases. In the first phase, water and ions like calcium and phosphate of the surrounding tissues reach the scaffold backbone, then the alloy reacts with the water to create Mg hydroxide. In the second phase, Mg hydroxide is gradually transformed into an amorphous calcium phosphate, which has abundant water content. Cracks infiltrated by cells, mainly macrophages, appear in the core as the material is getting reabsorbed [70]. As the magnesium scaffold broke down, it was also found to leave chemical byproducts that could lead to potential complications such as ectopic calcification in the surrounding tissue. About 95% of the magnesium alloy is resorbed within 12 months [71], which is accompanied by a rapid increase in the number of discontinuities found between 28 days and 1 year [71]. The first generation of the bioabsorbable metal scaffold (AMS-1) consisted of 93% magnesium and 7% rare earth elements with a strut thickness of 165 micron without drug. Although initial results showed good rate of patency, to avoid early loss of radial strength and vessel recoil and reduce late loss, further modifications were made to this magnesium-based BRS [72]. The Magmaris resorbable magnesium scaffold (RMS) (BIOTRONIK AG, Buelach, Switzerland), is coated with PLLA polymer to control the degradation speed and elutes sirolimus drug to reduce late loss.

Although the strut thickness of the current Magmaris RMS (150 µm) is similar to Absorb-BVS, pre-clinical studies from our group revealed higher thromboresistance of Magmaris RMS compared with Absorb-BVS and CoCr SES (Orsiro; BIOTRONIK AG, Buelach, Switzerland) (strut thickness, 60 µm), suggesting that magnesium itself may have antithrombotic properties [73]. In first-in-man clinical trial for Magmaris RMS, BIOSOLVE-II, including 123 patients (123 lesions), showed no definite nor probable scaffold thrombosis up to 12 months, and no target lesion failure beyond 6 months [72]. Long-term follow-up data and real world clinical evidence are still needed to confirm safety and efficacy for this device.

## 10. Future Perspectives

Stent biocompatibility includes multiple components: effecting simultaneous hemocompatibility, promoting rapid endothelial recovery and suppressing restenosis. Current metallic second-generation DES technology has very good clinical outcomes in acute- and middle-term follow-up periods, including very low rate of early thrombotic occlusion and restenosis. To overcome the long-term clinical drawback of permanent metallic DES (e.g., continued climbing up of target lesion revascularization with time, lack of adaptive remodeling, abnormal vasomotion), a totally bioabsorbable system still remains appealing in concept. However, unless comparable short- and middle-term clinical outcomes are able to be obtained, as is currently available for metallic DES, the theoretical long-term advantages for BRS cannot be guaranteed. Improved short and mid-term outcomes will only be possible with a reduction of strut thickness and vessel wall coverage area while maintaining radial strength. Minimizing recoil, early dismantling, and the inflammatory response during degradation process are essential for improving the vascular responses to BRS implantation. Material innovation is needed to overcome the current issues.

## 11. Disclosure

CVPath Institute has received institutional research support from 480 Biomedical, Abbott Vascular, ART, Biosensors International, BIOTRONIK, Boston Scientific, Celonova, Claret Medical, Cook Medical, Cordis, Edwards Lifesciences, Medtronic, MicroPort, Microvention, OrbusNeich, ReCore, SINO Medical Technology, Spectranetics, Surmodics, Terumo Corporation, W.L. Gore and Xeltis.

## Figures and Tables

**Table 1:** Composition of stent alloys (wt. %).

Materials	Fe	Co	Cr	Pt	Ni	W	Mo	Mn	Ti	Mg	Ir
**316L SS**	63	-	18	-	14	-	2.6	<2.0	-	-	-
**CoCr (L605)**	3	52	20	-	10	15	-	1.5	-	-	-
**CoCr (MP-35N)**	<1.0	34	20	-	35	-	9.75	<0.15	<1.0	-	-
**PtCr**	37	-	18	33	9	-	2.6	<0.05	-	-	-
**Titanium**	-	-	-	-	-	-	-	-	90–100	-	-
**Nitinol**	-	-	-	-	55	-	-	-	45	-	-
**Mgalloy**	-	-	-	-	-	-	-	-	-	93.6	-
**Pure iron**	99.8	-	-	-	-	-	-	-	-	-	-
**PtIr (90Pt/10Ir)**	<0.015	-	-	90	-	-	-	-	-	-	9.5–10.5

Co: cobalt, Cr: chromium, Fe: iron, Ir: iridium, Mg: magnesium, Mn: manganese, Mo: molybdenum, Ni: nickel, Pt: platinum, SS: stainless steel, Ti: titanium, W: Tungsten. Modified permission obtained from Foin, N.; et al. Int. J. Cardiol. 2014 [3].

**Table 2:** Material properties for metallic alloys and common biodegradable polymers.

Materials	Density (g/cm^3^)	Elastic Young’s Modulus (Gpa)	Tensile Strength (Mpa)	Elongation at Break (%)	Corrosion Resistance	Visibility	Biocompatibility	Low Recoil	Biodegradability (Months)
**316L SS**	8	193	670	48	+	+	+	+	−
**CoCr (L-605)**	9.1	243	>1000	>50	+	+	+	+	−
**CoCr (MP-35N)**	8.43	233	930	45–60	+	+	+	+	−
**PtCr**	9.9	203	834	45	+	+ +	+	+	−
**Nitinol**	6.45	40	800–1200	12–25	+	+	+	+	−
**Pure iron**	7.8	150	210	40	−	+	+ −	+	>12
**Fe-35MN**	7.6	235	530	32	−	+	−	n/a	>12
**Mg (WE43)**	1.83	40–130	280	6.8	−	+	+ −	+	1–3
**PLLA**	1.2–1.4	2.7–4.0	40–65	2–6	n/a	−	+	+ −	18–36
**PDLA**	1.8	1.0–3.5	40–55	2–6	n/a	−	+	+ −	12–16
**PGA**	1.5	6.0–7.0	90–110	1–2	n/a	−	+	+ −	4–6
**PCL**	1.1	0.2–0.4	25–35	>300	n/a	−	+	+ −	24–36
**PLGA (85 L/15 G)**	1.3	2.0–4.0	40–70	2–6	n/a	−	+	+ −	12–18
**PDLGA (50 DL/50G)**	1.2–1.3	2.0–4.0	40–50	1–4	n/a	−	+	+ −	1–2

Co: cobalt, Cr: chromium, Fe: iron, Mg: magnesium, PCL: poly-ε-caprolactone, PDLA: poly-d-lactic acid, PDLGA: poly(d,l-lactide-*co*-glycolide), PGA: polyglycolic acid, PLLA: poly-l-lactic acid, PLGA: poly(lactide-*co*-glycolide), Pt: platinum, SS: stainless steel. Permission obtained from Foin, N.; et al. Int. J. Cardiol. 2014 [3].
